# On Theoretical Models of Gene Expression Evolution with Random Genetic Drift and Natural Selection

**DOI:** 10.1371/journal.pone.0007943

**Published:** 2009-11-20

**Authors:** Osamu Ogasawara, Kousaku Okubo

**Affiliations:** Center for Information Biology and DNA Data Bank of Japan, National Institute of Genetics, Mishima, Shizuoka, Japan; Washington University, United States of America

## Abstract

**Background:**

The relative contributions of natural selection and random genetic drift are a major source of debate in the study of gene expression evolution, which is hypothesized to serve as a bridge from molecular to phenotypic evolution. It has been suggested that the conflict between views is caused by the lack of a definite model of the neutral hypothesis, which can describe the long-run behavior of evolutionary change in mRNA abundance. Therefore previous studies have used inadequate analogies with the neutral prediction of other phenomena, such as amino acid or nucleotide sequence evolution, as the null hypothesis of their statistical inference.

**Methodology/Principal Findings:**

In this study, we introduced two novel theoretical models, one based on neutral drift and the other assuming natural selection, by focusing on a common property of the distribution of mRNA abundance among a variety of eukaryotic cells, which reflects the result of long-term evolution. Our results demonstrated that (1) our models can reproduce two independently found phenomena simultaneously: the time development of gene expression divergence and Zipf's law of the transcriptome; (2) cytological constraints can be explicitly formulated to describe long-term evolution; (3) the model assuming that natural selection optimized relative mRNA abundance was more consistent with previously published observations than the model of optimized absolute mRNA abundances.

**Conclusions/Significance:**

The models introduced in this study give a formulation of evolutionary change in the mRNA abundance of each gene as a stochastic process, on the basis of previously published observations. This model provides a foundation for interpreting observed data in studies of gene expression evolution, including identifying an adequate time scale for discriminating the effect of natural selection from that of random genetic drift of selectively neutral variations.

## Introduction

It has long been hypothesized that phenotypic evolution is more often based on evolutionary changes in gene expression regulation than on sequence changes in proteins [1]. Prompted by this hypothesis and the advent of genome-wide gene expression profiling techniques, an increasing number of studies have investigated the pattern of evolutionary change in gene expression profiles and the evolutionary forces governing the process.

Recently, it has become evident that heritable variations in the mRNA abundance are commonly seen in a variety of species, such as yeast [2]–[5], *Drosophila* [6]–[10], mice [11], [12], and humans [13]–[16], along with variations between species [7], [9], [17]–[25]. This has raised the question of how those variations are maintained in populations and what evolutionary forces affect the pattern of variations within and among species. In particular, the main conflict between researchers over gene expression evolution is the relative contribution of random genetic drift and natural selection to evolutionary changes in mRNA abundance. Some researchers have argued that the majority of evolutionary changes in the mRNA abundance is selectively neutral and likely to be of little or no functional significance (neutral hypothesis) [17], [18], [21], [22], [24]–[26], while others have argued that natural selection has a substantial effect on gene expression evolution [7], [23]. According to Whitehead and Crawford (2006) [30], among studies which provide quantitative estimates, the proportion of genes which are supposed to be subject to stabilizing selection, for example, varies from 7% to 100%. In a comparison of populations of the marine killifish *Fundulus* adapting to different habitat temperatures, much of the variation in expression level was correlated with phylogeny regardless of the habitat temperature they adapted to [17], [18], [24], [25]. This result can be explained by the neutral hypothesis. On the other hand, Lemos et al. (2005) [23] argued that more than 96% of genes were subject to stabilizing selection in primates, rodents, and *Drosophila* lineages by using the mutation-drift equilibrium model [27]–[29] in which the variance in expression levels of a given gene among species was scaled by the divergence time.

As for the cause of this conflict, Whitehead and Crawford (2006) [30] suggested that the linearity between gene expression divergence and phylogenetic distance, which is expected from the neutral hypothesis, might be lost when the divergence time becomes sufficiently large, and that this might confuse the analysis. Therefore they pointed out that it is important to identify an adequate time scale for discriminating the effect of natural selection from that of random genetic drift of selectively neutral variations. In order to address this problem, it is crucial to construct a neutral model which can predict the long-run behavior of evolutionary changes in mRNA abundance.

A neutral model of gene expression evolution was first proposed by Khaitovich and coworkers (2004) [22]. They constructed this model based on the observation that gene expression divergence increases proportionally with divergence time in lineages of primates and rodents, which is termed as a “clock-like” accumulation of gene expression divergence. This observation can be explained from the assumption that mutations cause changes in relative amounts of expression levels irrespective of gene function [22], [26]. However, since those studies were confined mainly with relatively short terms of gene expression evolution, such as between humans and chimpanzees, the long-run behavior of the neutral model of gene expression evolution has not been well studied.

To investigate long-run behavior, in this study we focused on a property of the distribution of mRNA abundance. As soon as genome-wide gene expression profiling techniques were developed, it was revealed that there is a common tendency in the distribution of mRNA abundance: a few genes are expressed intensely and most genes are expressed at quite low levels. It is now known that this distribution can be well described by Zipf's law [31] (or its mathematical equivalent, called the power law) from vertebrates to lower eukaryotes [32]–[37]. This law states that there is a relationship between the mRNA abundance (*f* [copy/cell]) and its abundance rank (*r*) represented by 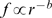. The exponent *b* is the absolute value of the slope in a logarithmic rank-frequency plot. It is remarkable that the value of exponent *b* is near 1.0 in most normal tissues. (More specifically, the exponent *b* is near 1.0 in normal tissues composed primarily of a homogeneous population of differentiated cells, such as liver and muscle. In other normal tissues composed of a mixture of different types of cells, including brain, testis, and kidney, the exponent *b* tends to be slightly lower than 1.0, as expected [35]).

Here it should be noted that if evolutionary forces affect the mRNA abundance of each gene, they inevitably affect the distribution of it. Moreover, the distribution of mRNA abundance can be expected to be generated by the long-term effect of evolutionary forces on gene expression regulation. Therefore, if most evolutionary changes in the mRNA abundance can be explained by the neutral hypothesis, then the above two mentioned phenomena, namely the clock-like accumulation of gene expression divergence and Zipf's law of the transcriptome, should be explained with the same neutral model. Indeed, Ogasawara et al. (2003) [35] suggested that Zipf's law of the transcriptome might originate from a process which is quite similar to the neutral model introduced by Khaitovich et al. (2004) [22]. However, as pointed out in this study, the previous model cannot explain the uniformity of the exponent *b*, which suggests that long-run behavior of previous models is not consistent with observations. In this study we propose refined models which can explain the two phenomena simultaneously by adding a few cytological constraints to the previous models.

## Results

### A Neutral Model of Gene Expression Evolution and the Genesis of Zipf's Law-Like Distribution in mRNA Abundance

Consider the mRNA abundance *f_i_*(*t*) (number of mRNA molecules in a cell) of gene *i* in a given cell type at generation *t*. We regard *f_i_*(*t*) as a heritable quantitative trait whose value is determined solely by the genetic effect, and we do not take account of the fluctuation caused by physiological and environmental factors. We assume that mRNA abundance *f_i_*(*t*) is affected by mutations with proportionality to the abundance before mutation. This can be expressed as follows:
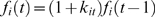
where *k_it_* is a mutually independent and identically distributed (iid) random variable with mean 0 and variance 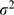, and is also statistically independent of *f_i_*(*t*). This assumption means that the probability of evolutionary change, from say 1.0 copy/cell to 2.0 copies/cell, is equal to the probability of change from 10 copies/cell to 20 copies/cell, and not to the probability from 10 copies/cell to 11 copies/cell. The neutral model of gene expression evolution introduced by Khaitovich et al. (2004) [22] is also based on essentially the same idea.

In this study, we refer to the model assuming that evolutionary changes in mRNA abundance are irrelevant to the function of the proteins as neutral model of gene expression evolution. On the other hand, we refer to the model assuming that the range of evolutionary changes is confined depending on the function of the protein as the natural selection model of gene expression evolution. We use the terminology from previous studies [22], [26], but it should be noted that the neutral model of gene expression evolution and Kimura's neutral model of molecular evolution are different concepts. Kimura's neutral theory asserts that the majority of DNA sequence polymorphisms observed within a species has no effect on the fitness, because the majority of harmful mutations are eliminated from the population by negative selection. Therefore it is obvious that both the natural selection model and the neutral model of gene expression evolution do not conflict with Kimura's neutral model of molecular evolution.

Assuming that the absolute value of *k_it_* is small compared with 1, we approximate from the Taylor expansion of 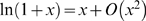 that
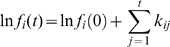



**Figure 1:**
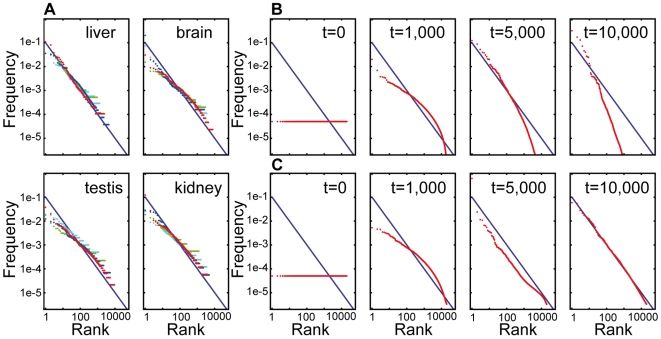
Log (rank) versus log (frequency) plot for mRNA abundance. (A) The mRNA abundance of human (red), mouse (green), chicken (blue), and *X. laevis* (light blue). (B) Result of the Monte Carlo simulation, *L* = 0.0. (C) Result of the Monte Carlo simulation, *L* = 1.0. At the initial state, the mRNA abundance of all genes is set to *M*/*N*. Other model parameters of (B) and (C) are: *M* = 300,000, *N* = 20,000, and *s_k_* = 0.05. The line shows *y* = *0.1/x*

Since we assume that *k_ij_* (*j* = 1, 2,…, *t*) is iid random variables, according to the central limit theorem, 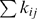 will be approximately a normal distribution with mean 0 and variance *s_k_^2^* = 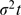 when 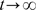. Therefore, 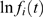 is asymptotically normally distributed and hence *f_i_*(*t*) is lognormally distributed. In general, a positive random variable *Z* is said to be lognormally distributed with two parameters, mean 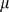 and variance *s^2^*, if 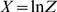 is normally distributed with them. It is known that when *s^2^* is sufficiently large, the lognormal distribution would appear almost linear on both a log-log plot of the probability density function (power-law plot) and on a Zipfian plot (Figure 1). This is the model of the genesis of Zipf's law of the transcriptome suggested by Ogasawara [35], and this process is generally known as the Gibrat process or the law of proportionate effect which was first introduced as an explanation of firm size distribution [38]–[41].

However, since the variance of the resultant distribution of 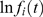 is given by 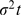, it increases with generation *t* without limit. Consequently, the exponent *b* also increases with *t* without limit (Figure 1 and Figure S1). Obviously this situation is unrealistic because there is no reason for many species to have an appropriate value of *t* to make *b* be near 1.0 concurrently. Therefore this model is inconsistent with observations.

We refined this previous model by adding a few cytological boundary conditions. First, we assume that the total number of mRNA molecules (*M*) in a given cell is constant throughout a specific evolutionary process. Secondly, the number of expressed genes (*N*) in a given cell type is constant throughout this evolutionary process. In typical mammalian cells, *M* and *N* are estimated to be 300,000–500,000 and 10,000–30,000, respectively [42]–[44]. This assumption implies that the mRNA abundance in a cell is determined by the relative, instead of absolute, affinity of the gene regulation proteins and the *cis*-elements among the genome, since those proteins would “choose” their target from a collection of *cis*-elements in the gene expression process. This corresponds to the normalization step in this model (see Materials and Methods).

It should be noted that by assuming the maximum capacity of mRNA in a cell (M), a limitless increase in the variance of the mRNA abundance by the Gibrat process leads most genes to decrease their mRNA abundances without limit. That is, most genes will lose their gene expression ability eventually (Figure 1 and Figure S1). Obviously this seems unrealistic and contradicts the assumption of the constancy of *N*. To avoid this, we make a third assumption that an individual who loses gene expression of functional genes will be eliminated from the population by negative selection. We formulate this assumption by prohibiting the mRNA abundance of each gene from being lower than a certain limit *L*; in the formula, 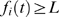 for any *i* and *t* (Materials and Methods). Some genes may have a different lower limit *L* from other genes according to their function; however, we ignore these as minor cases for the purpose of modeling gene expression evolution.

We next investigated the long-run behavior of the refined model by a series of Monte Carlo simulations. The simulations indicate that the process approaches the steady-state, Zipf's law-like distribution regardless of the initial state distribution when model parameters are set to the typical value of mammalian cells (*M* = 300,000, *N* = 20,000), and the lower limit of expression level *L* is set to 1.0 [copy/cell] (Figure 1C). The consistency of the model (with *L* = 1.0, *M* = 300,000, and *N* = 10,000) and data has been tested by the generalized chi-square goodness-of-fit test [45]. We use the EST-based gene expression profiles of livers of human, mouse, chicken, and *Xenopus laevis*, since liver tissue is composed primarily of a homogeneous population of differentiated cells. Estimating the discrepancy measure *d* from human liver EST profiles (*d* = 0.0268), the P-values of the generalized goodness-of-fit tests of human, mouse, chicken, and *X. laevis* were 1.0 (n = 2,384), 0.997 (n = 762), 0.794 (n = 935), and 0.999 (n = 1304), respectively (*df* = 4). Even with a rather conservative assumption that the relative error in the EST profiles is only 3% for each bin (*d* = 9.0×10^−4^), P-values of human, mouse, chicken, and *X. laevis* were 0.68, 0.46, 0.0025, and 0.10, respectively (see Materials and Methods). In conclusion, the model is not rejected by the data. The relatively small P-value of the chicken data in the latter case can be readily explained by the measurement error of EST-based profiling.

**Figure 2:**
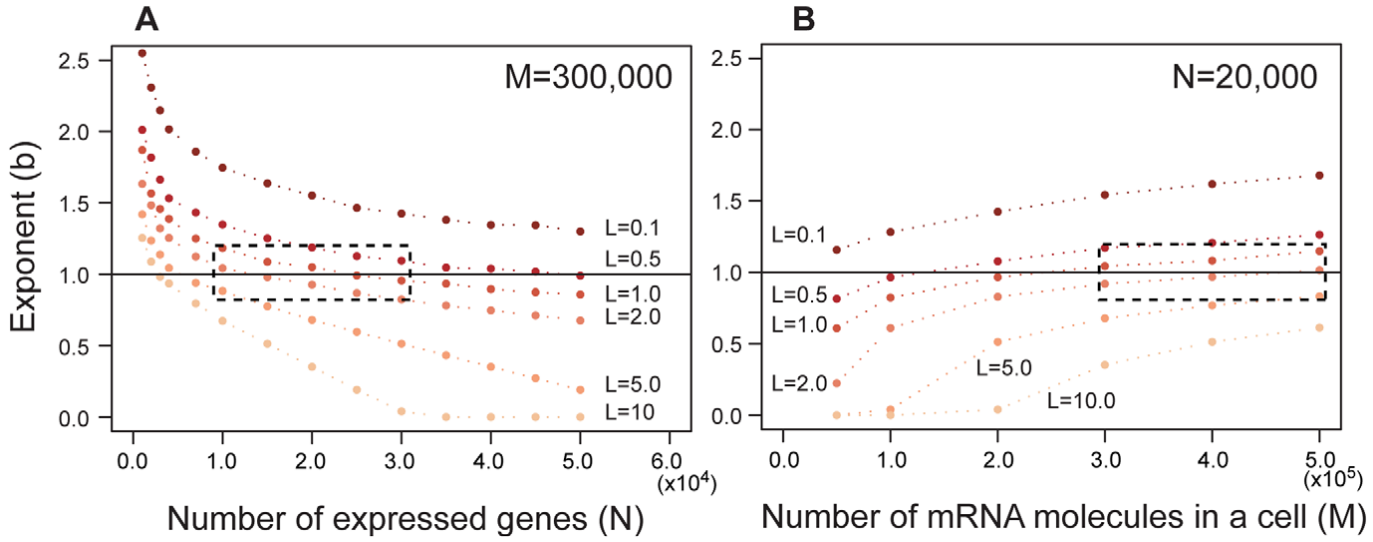
Relationship between model parameters *L*, *M*, *N*, and exponent *b*. (A) *M* is fixed to 300,000. (B) *N* is fixed to 20,000. Each point shows the exponent *b* averaged over 50 repetitions of the simulation, where *s_k_* = 0.05 and *t* = 10,000. At the initial state, the mRNA abundance of all genes was set to *M*/*N*. The rectangle shows the range of the parameters in typical mammalian cells.

We also show by simulation that the standard deviation of the random coefficient *k_it_* has little effect on the form of the steady-state distribution, as expected from the central limit theorem, but that it affects the evolutionary rate. That is, the number of generations required for convergence increases as the standard deviation of *k_it_* decreases (Figure S2). This result implies that the exponent *b* is solely determined by the model parameters *L, M*, and *N*. Therefore, we examined the relationship between the values of *L, M, N*, and the resultant exponent *b* of the steady-state distribution (Figure 2). The result of the Monte Carlo simulation shows that exponent *b* is much more susceptible to *L* but hardly affected by *M* and *N*. The exponent *b* takes a value close to 1.0 over a wide range of *M* and *N* if and only if *L* is near 1.0 copy/cell.

From this result, the uniformity of exponent *b* can be explained as follows. The number of expressed genes *N* and total number of mRNA molecules in a cell *M* may differ among cells, but those parameters have little effect on the value of *b*. On the other hand, the lower limit *L* might be common across a variety of cells, because if *f_i_*(*t*) becomes under 1.0 copy/cell by mutation, that means the transcript can no longer exist at every moment in the cytoplasm. This situation would, in effect, be similar to the gene being lost; hence, it would be subject to negative selection. Therefore *L* would have a similar value across a variety of cells, and consequently *b* would be the same across a variety of cells.

### Loss of Clock-Likeness in the Long-Run Behavior of Gene Expression Divergence under the Neutral Model

It is well known that Zipf's law-like distributions can be generated by a variety of stochastic processes [40], [41], [46], [47]. Those processes include the law of proportionate effect which is described above, the theory of breakage studied by Kolmogoroff [48], the preferential attachment process studied by Yule [49] and Simon [50], the optimization theory of the genesis of Zipf's law studied by Mandelbrot [51], and the distribution of powers and products of normal variables studied by Haldane [52]. On the other hand, some people even believe the superstition that a Zipf's law-like distribution can be generated “without cause”. Needless to say, this belief is not true. The truth is that in some phenomena the cause of the genesis of the distribution is not known, and in some phenomena the cause is trivial and rather disappointing. Regardless of how many models were introduced previously, the law of proportionate effect is currently a good starting point available for the explanation of the genesis of the Zipf's law-like distribution in mRNA abundance, since it is the basis for the model of the clock-like accumulation of gene expression divergence [53]–[55]. It should be recalled that if evolutionary forces affect the mRNA abundance of each gene, they inevitably affect their distributions. Therefore, the explanations of the clock-like accumulation and the Zipf's law-like distribution must be based on the same set of mechanisms.

Both the distribution of mRNA abundance in a cell (A) and the gene expression divergence (B) are solely determined from the gene expression profiles (C), where a gene expression profile is a list of the mRNA abundance of each gene *f_i_*(*t*), where *i* = *1*, …, *N*, at a given *t*. Namely, (A) and (B) are two different features of the same source (C). Therefore, if a model insists that it can explain (A) through a formulation of (C), the model should explain (B) simultaneously, and vice versa. One may say that *f_i_*(*t*) can change among species without any effect on the overall distribution of *f_i_*(*t*) in a cell; say *f_1_*(0) = 1.0, *f_2_*(0) = 2.0 to *f_1_*(1) = 2.0, *f_2_*(1) = 1.0. Even in such cases, the source of the constraints which force the distribution into being unchanged should be explained from the time-course of *f_i_*(*t*) formulated by the model.

**Figure 3:**
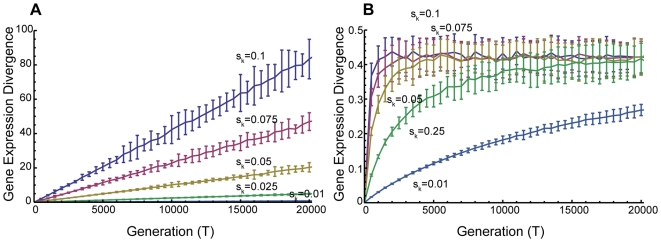
Time development of gene expression divergence. Each point shows the gene expression divergence averaged over 100 repetitions of the simulation. (A) *L* = 0.0. (B) *L* = 1.0. Other model parameters are: *M* = 300,000 and *N* = 20,000. Initial distribution (*t* = 0) of the simulations follows Zipf's law.

Therefore, we examined whether the refined model, which has been introduced to explain the Zipf's law-like distribution, can also regenerate another independently found phenomenon—the clock-like accumulation of gene expression divergence discovered by Khaitovich et al. (2004) [22]. The Monte Carlo simulation showed that when the previous model (specifically, in the case of *L* = 0.0, *M* = 300,000, and *N* = 20,000) is assumed, it follows that gene expression divergence increases linearly without limit (Figure 3A). On the other hand, the refined model (*L* = 1.0 copy/cell) predicts that at first gene expression divergence increases linearly, then the increasing rate decreases gradually, and eventually converges to an upper limit near 0.4, although it exhibits substantial stochastic fluctuations (Figure 3B). The upper limit is found to be unaffected by the standard deviation *s_k_* of the distribution of *k_it_* (Figure 3B), and slightly affected by *M* and *N* (data not shown), similar to the case of the genesis of Zipf's law-like distribution. This prediction of the presence of the upper limit is one of the discriminating features of the refined model which can be used as a criterion for its falsification.

**Figure 4:**
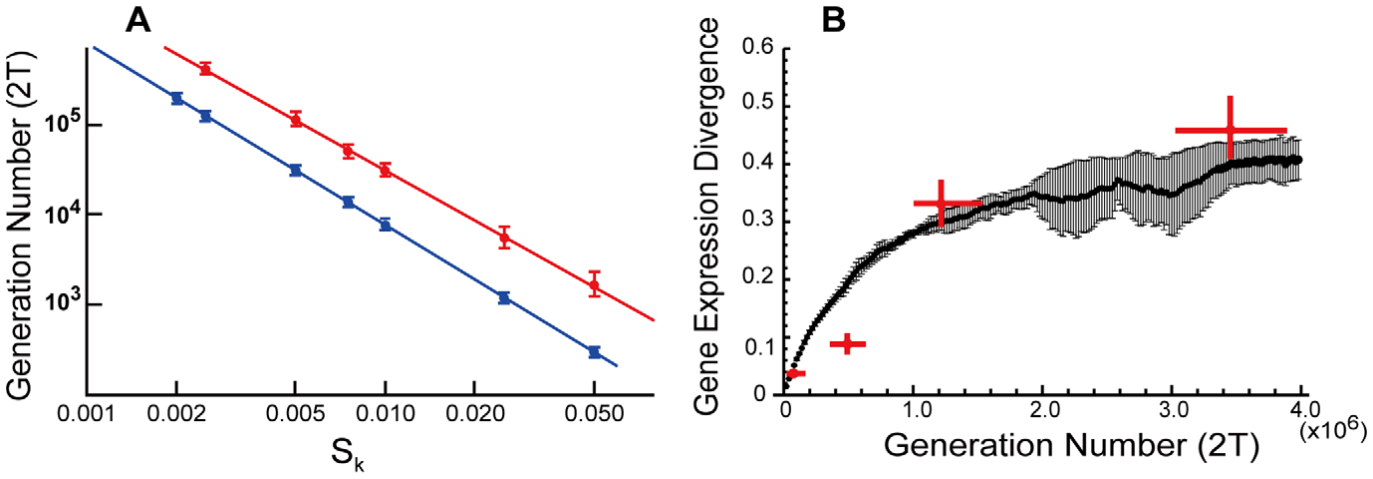
Time development of gene expression divergence of primate brains. (A) The number of generations required for gene expression divergence to be 0.3. Each point is the mean value averaged over 100 repetitions. The red line is L = 1 and the blue line is L = 0. (B) Time development of gene expression divergence. Red points are the gene expression divergence between humans and other primates reported by Khaitovich et al [22]. Black points are the result of the simulation averaged over 10 repetitions, and error bars represent standard deviation. In the both panels, model parameters are: *L* = 1.0, *M* = 300,000, and *N* = 20,000. Initial distribution (*t* = 0) of the simulations followed Zipf's law.

The time-course of gene expression divergence reported in Khaitovich et al. (2004) [22] exhibits signs of an upper limit. According to the neutral theory [56] and the generation time effect hypothesis [57]–[59], the evolutionary rate is a function of the number of generations rather than chronological time. From a human-orangutan comparison, the divergence increases about 0.3 in about 2T = 1.2 million generations, but from human-orangutan and human-macaque, the divergence increases only by about 0.1 in about additional 2.3 million generations (Figure 4) [22]. Here we assume that divergence of human-chimpanzee, human-orangutan, and human-macaque takes approximately some 6, 13, and 23 million years, respectively [60], and that average generations in human, chimpanzee, orangutan, and macaque are 28, 22, 20, and 11.4 years, respectively [61], [62].

In addition, an upper limit can be clearly seen in the time-course of gene expression divergence based on a subset of genes which has the largest evolutionary rate (25% of genes that has the largest human variation among 2,926 genes measured). From a human-chimpanzee comparison to human-orangutan comparison, the divergence increases about 0.44, but from human-orangutan to human-macaque, the divergence increases only about 0.06 [22]. Therefore, at present, the refined model appears to be more consistent with observations than previous models. Moreover, our model supports the suggestion by Whitehead and Crawford (2006) [30] that the inconsistency of results from several previous studies is caused by the loss of linearity between gene expression divergence and generation over the long-run. Therefore our model might be used for predicting appropriate time scales for distinguishing the effects of random drift and natural selection in the primate lineage.

The estimate of standard deviation *s_k_* under the neutral model can be obtained from the expression divergence data of primates [22] as follows. Monte Carlo simulations indicate that the number of generations needed for gene expression divergence to reach a given value, say 0.3 (human-orangutan), decreases rapidly and that the evolutionary rate of the previous model (*L* = 0.0) is generally larger than that of the refined model (*L* = 1.0). The relationship between *s_k_* and the number of generations is well fitted to 2T = 4.79 s_k_^−2.02^ (*L* = 1.0) and 2T = 1.67 s_k_^−1.99^ (*L* = 0). From this formula, the standard deviation *s_k_* of the primate lineage was estimated from the observations of Khaitovich et al. (2004) [22] as about 2.15×10^−3^ under the neutral model (Figure 4).

### Models of Gene Expression Evolution with Natural Selection

In the previous sections, we discussed neutral models of gene expression evolution in which evolutionary changes in mRNA abundances are assumed to be irrelevant to the functions of the proteins. However, since it is evident that adequate regulation of the mRNA abundance is a prerequisite for efficient protein function, it is necessary to extend the model to account for the effect of natural selection which optimizes the mRNA abundance.

As we mentioned previously, the distributions of mRNA abundance in cells are similar in a variety of species, including mammals, insects, plants, and lower eukaryotes. It seems that this distribution is not easily explained from a natural selection model of gene expression evolution, because these species having different sets of genes are adapted to different environments and different ways of life. If those genes have their own optimum mRNA abundance *(μ_i_)* according to their functions, then the observed distribution of mRNA abundance in cells would differ among the various species. There is no reason that each of the optimum values *{μ_i_} (i = 1,…,N)* follows Zipf's law-like distribution. Rather, *{μ_i_}* can take any shape, even one far from the Zipf's law-like distribution depending on the functions of the proteins, since distantly related species, such as insects and plants, have different sets of genes and are adapted to different environments with different ways of life. The problem here is how these two seemingly contradictory phenomena can be explained simultaneously from a unified model.

First, we examined whether the steady-state distribution can be altered, assuming the optimum mRNA abundance for each gene. Since Zipf's law-like distributions are observed in a variety of phenomena, one may expect that this type of distribution is so robust that it is not altered by additional factors such as natural selection. To examine this hypothesis, we undertook the Monte Carlo simulation with random mating populations with finite population size comprised of sexually reproducing diploid organisms. We assume that the mRNA abundance of each locus is determined by the average effect of two genes on the locus, and that fitness of each individual is determined by the mRNA abundance of a given set of genes.

**Figure 5:**
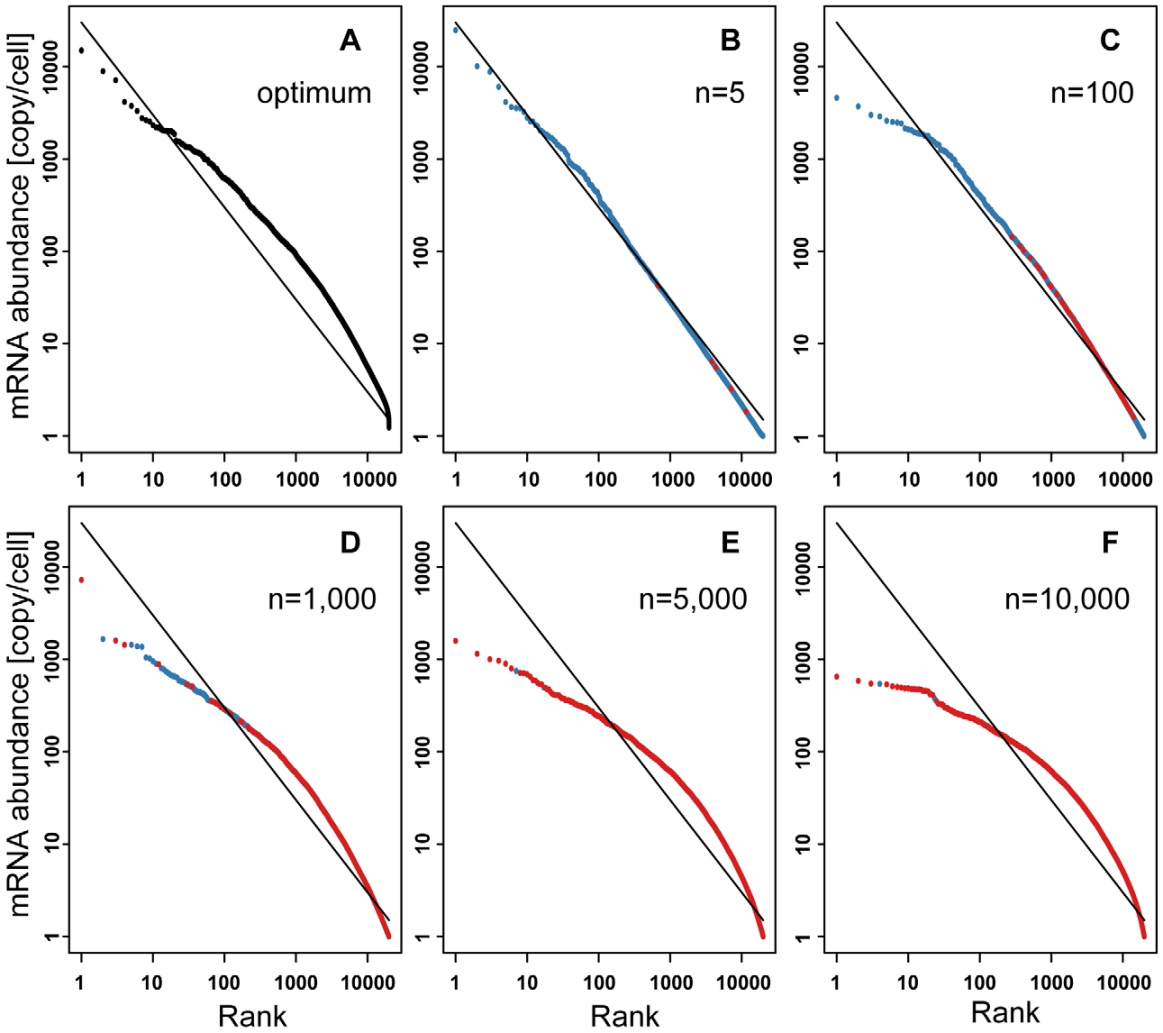
Distribution of mRNA abundance under the optimum mRNA abundance model. (A) Optimum value distribution dassumed in the Monte Carlo simulation. (B–F) Steady-state distributions of mRNA abundance where *n* genes are subject to natural selection. Red points represent genes undergoing natural selection. Blue points are neutral genes. Model parameters are: *L* = 1.0, *M* = 300,000, *N* = 20,000, and *s_k_* = 0.01. Population size (*N_e_*) is 100. *t* = 50,000. The selection intensity is 1.67.

At present, there is insufficient information on “the distribution of the optimum mRNA abundances” for simulation. We can only conjecture that there is no reason for a variety of species to have an identical optimum value distribution. Therefore, we assume that the optimum value distribution has a slightly distorted shape from the Zipf's law-like distribution with the optimum value distribution given as *10^X^*+1, where *X* is a random variable having a skewed normal distribution (Figure 5). Then we examined whether the steady-state distribution converges to the Zipf's law-like distribution.

The Monte Carlo simulation demonstrated that the steady-state distribution is readily altered by the effect of natural selection, although only when a small number of genes (about 0–100) is assumed to be at their optimum did the process converge into a Zipf's law-like distribution (Figure 5). It should be noted that in these simulations the selection intensity (defined as the reciprocal of the proportion of individuals selected from a population to be used as parents) was the same across different values of *n*, the number of loci undergoing the natural selection (Materials and Methods).

This result showed that the Zipf's law-like distribution was not robust against the effect of natural selection under the absolute optimum value model. This suggests that it is not easy to explain the universality of the Zipf's law-like distribution under the absolute optimum value model without assuming that the optimum value distribution itself follows Zipf's law. However, there is no reason to think that this additional assumption holds. Rather, if there exists an optimum value distribution, it seems natural that distantly related species would have different optimum value distributions as mentioned above.

Bedford and Hartl (2009) [55] reported that, in several *Drosophila* species, average pairwise variance in the mRNA abundance as they defined it was saturated at around 0.3. They pointed out that the variance is expected to equal 1.0 under the neutral model of gene expression. Indeed, the expectation of our refined neutral model is also 1.0. For an explanation of this observation, Bedford and Hartl (2009) [55] proposed an evolutionary model based on the Ornstein-Uhlenbeck (OU) process described as following a stochastic differential equation:
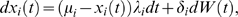
where, *x_i_*(*t*) = *log_2_f_i_*(*t*) and *f_i_*(*t*) is the mRNA abundance of gene *i* at time *t*, and μ_i_ is the log of optimum mRNA abundance of gene *i*. W is the standard Brownian motion (also called a Wiener process), and λ_i_ and δ_i_ are positive constants corresponding to the strength of natural selection and random genetic drift acting on gene *i*, respectively. In other words, Bedford and Hartl argued that evolutionary change in the mRNA abundance can be described by the balance between the drift of Brownian motion and natural selection pulling the mRNA abundance toward its optimum.

**Figure 6:**
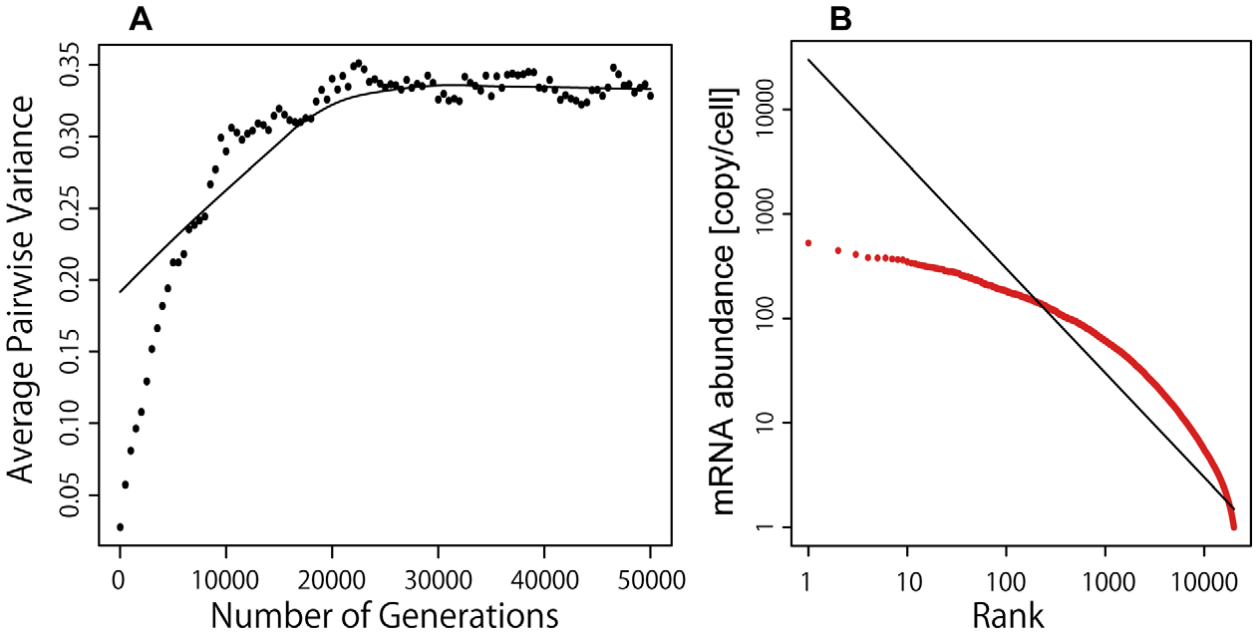
Optimum mRNA abundance model. (A) Time development of average pairwise variance. (B) Steady-state distributions of mRNA abundance. Model parameters are: *L* = 1.0, *M* = 300,000, *N* = 20,000, *s_k_* = 0.01, and *n* = 20,000. Population size (*N_e_*) is 100. *t* = 50,000. The selection intensity is 1.67.

However, since the Bedford-Hartl model assumed an optimum value μ_i_ (*i* = *1*, …, *N*) of the mRNA abundance for every gene, it is not easy to explain the Zipf's law-like distribution of mRNA abundance in cells from this model. In our simulation scheme, a selection intensity of 1.67 is necessary for constraining the Bedford's average pairwise variance to around 0.3; however, the steady-state distribution of mRNA abundance departs from the Zipf's law-like distribution under this selection intensity (Figure 6).

**Figure 7:**
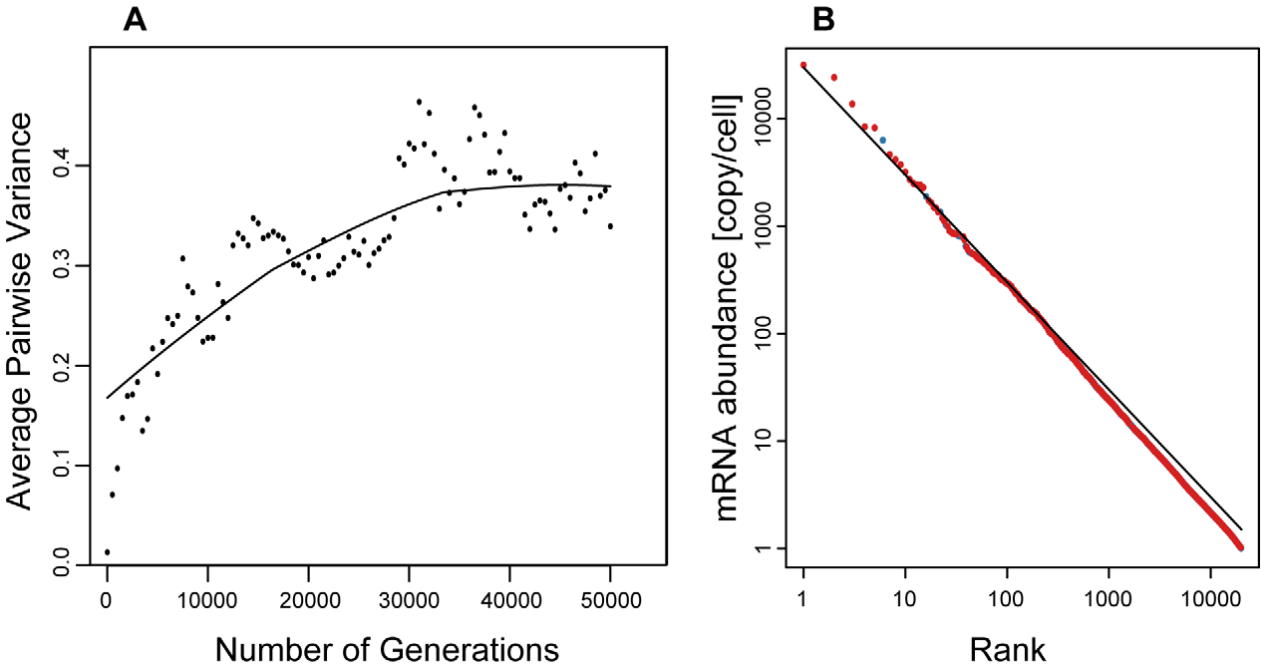
Relative abundance model. (A) Time development of average pairwise variance. Each point is mean value over 25 repetitions of the simulation. (B) Steady-state distributions of mRNA abundance. Red points represent genes undergoing natural selection. Blue points are neutral genes. Model parameters are: *L* = 1.0, *M* = 300,000, *N* = 20,000, *s_k_* = 0.01, and *n* = 500. Population size (*N_e_*) is 100. *t* = 50,000. The selection intensity is 5.0.

In order to reconcile this apparent inconsistency, we introduce another model of gene expression evolution. In this model, we assume that the genes have no intrinsic optimum mRNA abundance such as *μ_i_*. Instead, the mRNA abundance suitable for their function is determined by their relative relationship to the other genes, such as *f_i_>f_j_*. Such relationships would reflect the whole structure of gene regulatory pathways, and would thus be as complex. Here we assume that the relationships are *f_1_>f_2_>*…*>f_n_*, for simplicity. The Monte Carlo simulation demonstrated that this model can explain both the Bedford's average pairwise variance and the Zipf's law-like distribution simultaneously (Materials and Methods, Figure 7).

The behavior of the relative abundance model can be summarized as follows: the distribution of mRNA abundance in a cell is determined both by random genetic drift and by cytological constraints, and the rank of mRNA abundance of each gene is optimized by natural selection.

## Discussion

In this study, we introduce a novel, explicitly defined model of gene expression evolution. Our model assumes that (1) the mRNA abundance is affected by mutations proportional to the abundance before mutation; (2) the number of expressed genes and total number of mRNA molecules in a cell type is nearly constant throughout a specific evolutionary process; (3) the mRNA abundance of each gene has a lower limit near 1.0 copy/cell; and (4) the mRNA abundance of each gene (*f_i_*) suitable for its function is determined by its relative relationship to other genes, i.e., *f_i_>f_j_*.

A series of Monte Carlo simulations demonstrated that this model can explain the following three phenomena simultaneously: (i) the clock-like accumulation of gene expression divergence reported by Khaitovich (2004) [22]; (ii) the diminished upper limit of the average pairwise variance reported by Bedford (2009) [55]; (iii) the Zipf's law-like distribution of mRNA abundance in a wide variety of species.

Although the effect of cytological constraints on the time development of gene expression divergence has been predicted by Lemos et al (2005) [23], an explicit formulation of cytological constraints has not been given in previous works. The present work introduces a formulation of cytological constraints (assumptions 2 and 3) and a scheme of natural selection optimizing the mRNA abundance (assumption 4), based on the relationship between the evolutionary change in the mRNA abundance and the distribution of mRNA abundance in a cell.

One feature of this model is that locus-specific gene expression divergence should not increase linearly with generation number; on the contrary, it behaves like a random walk. Therefore the distribution of locus-specific gene expression divergence should exhibit a rather different and complex shape depending on the divergence time. On the other hand, Khaitovich's gene expression divergence was defined by the average value over all loci comprising the transcriptome, instead of being locus-specific. In this case, the divergence should increase linearly with generation time; that is, a clock-like accumulation of gene expression divergence. This is an essential difference between our model and an infinite site model of neutral polymorphisms of nucleotide sequences in which locus-specific divergence accumulates proportionally with generation time.

Although our model currently focuses on describing the evolutionary process of a single cell for simplicity, this model might also be applicable for describing the gene expression evolution of multicellular organisms. The results of genome-wide gene expression profiling of higher eukaryotes, including humans, indicate that most genes can be clearly classified into two well-known categories, housekeeping genes and tissue-specific genes, which are expressed in a small number of cell types. This suggests that the distribution of mRNA abundance among different types of cells is not independent, but rather that the mRNA abundance of housekeeping genes in one cell type evolves to be larger, and would therefore become larger in other cell types in most cases. On the other hand, in the case of tissue-specific genes, the evolution of the mRNA abundance in the cell type where the gene is expressed would not affect the mRNA abundance of other cell types, because by definition it would be confined to the smallest expression level. The evolutionary change of the anatomical pattern of gene expression [63]–[67] is another interesting area to investigate, although for this purpose our model should be expanded for evaluation of the effects of gene duplication.

Several other models have been proposed to explain the Zipf's law-like distribution of mRNA abundance. Without exception, those models were based on the dynamics of mRNA synthesis and degradation in a cell, rather than evolutionary processes. Furusawa and Kaneko proposed a model which asserts that the distribution originates from the balance between the effect of up-regulating genes and down-regulating genes in the intracellular gene regulation network [33]. Kuznetosov assumed that the dynamics can be described by the birth-death stochastic process [34]. Similarly, Ueda et al. formulated this process as geometrical Brownian motion [37]. According to the formulation of any of those dynamic models, it follows that the expression level of every gene would fluctuate within the full range, namely from 0.0 copy/cell to the maximum value permitted, virtually at random. To avoid this consequence, those models must make an additional assumption that each gene has its own dynamic range of expression levels. This course of thinking would lead to the basic idea of the evolutionary model, where the gene expression level is determined by its genome.

## Materials and Methods

### Estimation of mRNA Abundance Based on EST Frequencies

We downloaded the UniGene data files of *Homo sapiens* (Build #210), *Mus musculus* (Build #170), *Gallus gallus* (Build #39), and *X. laevis* (Build #82) from the NCBI FTP site. With reference to the property table of cDNA libraries provided by the BodyMap-Xs database (http://bodymap.jp) [68], we selected cDNA libraries having the largest number of EST sequences among the libraries which were originated from pathologically normal tissues of adult organisms, and which did not suffer from experimental procedures such as normalization, subtraction, selection, and full-length cDNA enrichment. We estimated the mRNA abundance by dividing the number of EST sequences belonging to each UniGene cluster in each cDNA library by the total number of EST sequences in the cDNA library. The list of the dbEST library IDs we used in the analysis is as follows: liver 6989 (human), 2484 (mouse), 11,222 (chicken), 5540 (*Xenopus*); brain 18318 (human), 12634 (mouse), 15560 (chicken), 8910 (*Xenopus*); testis 18476 (human), 11976 (mouse), 15563 (chicken), 12882 (*Xenopus*); kidney 18374 (human), 7268 (mouse), 11220 (chicken), 11985 (*Xenopus*).

### Monte Carlo Simulation (1) Simple Stochastic Process

For the examination of the convergence to the Zipf's law-like distribution, we assumed that the mRNA abundance at the initial state (*t* = 0) was *f_i_*(*0*) = *M/N* for each gene (*i* = *1,2,* …, *N*). At the transition to the next generation, the mRNA abundance of each gene was altered by mutation following *f_i_'*(*t+1*) = *f_i_*(*t*) (*1+k_it_*), where *k_it_* was the normally distributed random variable. Then we normalized the mRNA abundance to 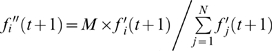 in order to grant 
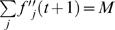
 at this time. For each gene *i*, if *f_i_″*(*t+1*)*<L* then the value was set to *f_i_″*(*t+1*) = *L.* Then we assigned *f_i_*(*t+1*) = *f_i_″*(*t+1*); that is, *f_i_*(*t+1*) is the mRNA abundance of gene *i* in the next generation.

For the time development of gene expression divergence, we assumed that the distribution of mRNA abundance at the initial state (*t* = 0) followed the Zipf's law-like distribution generated by the Monte Carlo simulation described above. We executed two independent simulations described above during the same number of generations (*T*), and we calculated the gene expression divergence defined by Khaitovich et al. between those two hypothetical species [22]. The computer program for the Monte-Carlo simulation was developed in our laboratory and written in Java version 1.6.0. The fitting procedure was executed using Mathematica version 6.0.

### Monte Carlo Simulation (2) Random Mating Population with Natural Selection

Using a random mating, finite, and constant size (*N_e_* = 100) population of diploid organisms, we assumed that every locus was inherited independently. We assumed that a given number of genes (*n*) were subject to natural selection, and that the other genes (*N*-*n*) were not subject to natural selection.

For the intensity of selection (defined as the reciprocal of the proportion of individuals selected from a population to be used as parents) to be the same across different values of *n*, we used the following selection schemes. We assumed that at the initial state (*t* = *0*), the mRNA abundance followed the Zipf's law-like distribution generated by the refined neutral model. The initial distribution was identical for all individuals. Here, *f_1_*(*0*)*>f_2_*(*0*)*>*…*>f_N_*(*0*) were satisfied. The mRNA abundance of each gene in each individual evolved by the process described above. The model parameters were: *L* = 1.0, *M* = 300,000, and *N* = 20,000.

In the optimum abundance model, we assumed that the optimum values distribution was given as *10^X^*+1, where *X* was a random variable having a skewed normal distribution with parameter *α* = 5 (Figure 5). We generated this random number sequence using an sn (the skew-normal and skew-t distributions) package of R statistics system version 2.8.0. The optimum values satisfied the inequalities *h_1_>h_2_>*…*>h_n_*, where *h_i_* was the optimum value of gene *i*. The relative fitness of an individual was given by the reciprocal of the sum of Euclidian distances of each gene, from the optimum values to the realized expression levels. Then a given number of individuals having the largest relative fitness survived to propagate the next generation.

In the relative abundance model, we assumed the optimum relationships to be *f_1_>f_2_>*…*>f_n_*. We tested the inequalities for all combinations of genes (*C*(*n,2*) = *n!/*((*n-2*)*!2!*) times), and we counted the number of inequalities satisfied in each individual. We used this count as the score of the individual, and a given number of individuals having the largest score survived to propagate the next generation. Since huge numbers of inequalities must be tested for all individuals in every generation, this simulation was very time consuming when we assume that *n* = *N* = *20,000*. Therefore we confined the number of genes undergoing natural selection to *n* = 500. The average pair-wise variance was calculated on the *n* genes undergoing the natural selection. For those simulations, we used Endeavor Pro7000 (Epson) Intel Core i7 3.20 GHz ×4 cores and SPARC Enterprise M8000 (Fujitsu) SPARC64 VII 2.52 GHz ×32 cores

### Generalized Chi-Square Goodness-of-Fit Test

The consistency of the model and the data were tested by the generalized chi-square goodness-of-fit test. A common problem of the standard Pearson's chi-square test is that because its power depends on sample size, small and unimportant departures from a specified reference distribution may be detected with large samples in general. The generalized chi-square goodness-of-fit test assumes that the observed distribution may contain a small discrepancy from the reference distribution that is eventually detected as the sample size increases [45]. The formula for the null hypothesis (*H_0_*) of the generalized chi-square test is *H_0_*: *d* = *d_0_, d_0_>0*, where *d* is the discrepancy measure defined as 
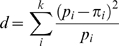
, 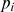 is the *i*-th cell probability given by the reference distribution 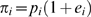, where *e_i_* is the relative error in the *i*-th cell, and *d_0_* is a constant given by the null hypothesis. Using those symbols, the null hypothesis of the Pearson chi-square test can be described as *H_0_*: *d* = *0*. When there is a discrepancy between the reference and observed distribution, the distribution of Pearson's chi-square statistics can be approximated by a non-central chi-square distribution with mean 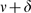, where 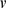 is the degree of freedom and 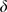 is the non-centrality parameter calculated by 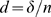, where *n* is the sample size.

We used the EST based gene expression profiles of livers of human, mouse, chicken, and *X. laevis*, since liver tissue is composed primarily of a homogeneous population of differentiated cells, and the EST frequency data can provide the estimate of the absolute mRNA abundance. (For other tissues primarily composed of a mixture of different kind of cells, we do not have enough parameters necessary for the goodness-of-fit test, such as the numbers and proportions of different kinds of cells and the degree of discrepancy of gene expression profiles among those types of cells.) Common logarithms of EST counts were binned into five bins (0–0.5, 0.5–1.0, 1.0–1.5, 1.5–2.0, and more than 2.0), because the gene expression profiles based on ESTs are very sparse sequences of discrete values of EST counts and the number of genes.

The reference distribution of tag frequencies was obtained from the Monte Carlo simulation with the model parameters *L* = 1.0, *M* = 300,000, and *N* = 20,000 as follows. The hypothetical gene expression profiles were generated by the Monte Carlo simulation (*s_k_* = 0.01, 100,000 generations), and we randomly chose 10,000 tags, and binned the result as described above. This trial was repeated 100 times and the averaged hypothetical tag frequency were used as the reference distribution of the goodness-of-fit test.

The relative error of each bin was estimated by the CV (standard deviation/mean) calculated from EST profile data of human liver (dbEST library IDs: 6989, 252, 12555). The CV of each bin was 0.037, 0.35, 0.11, 0.63, and 0.056, respectively.

## Supporting Information

Figure S1 Time development of hypothetical mRNA abundance generated by Monte Carlo simulations of the previous model (L = 0.0). Other model parameters were: M = 20,000, N = 300,000. The line shows y = 0.1/x.
(0.37 MB PDF)

Figure S2 Time development of hypothetical mRNA abundance generated by Monte Carlo simulations of the refined neutral model (L = 1.0) Other model parameters were: M = 20,000, N = 300,000. The line shows y = 0.1/x.
(0.39 MB PDF)
