# Supplementary figures and images for: On Theoretical Models of Gene Expression Evolution with Random Genetic Drift and Natural Selection
Source: PLoS One. 2009 Nov 20;4(11):e7943. doi: 10.1371/journal.pone.0007943 (PMC2776274; doi:10.1371/journal.pone.0007943)

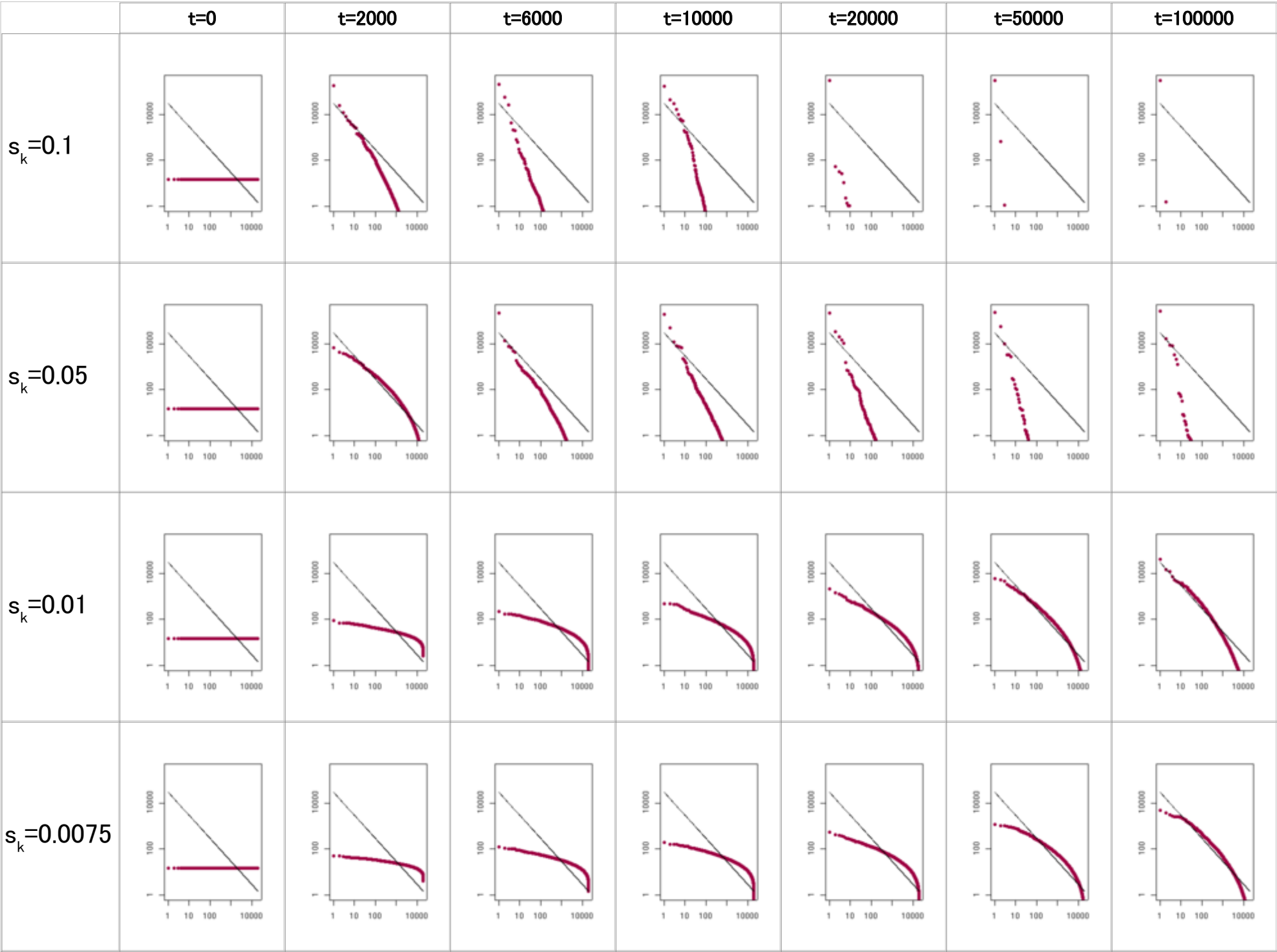

Supplement: Figure S1 — Time development of hypothetical mRNA abundance generated by Monte Carlo simulations of the previous model (L = 0.0). Other model parameters were: M = 20,000, N = 300,000. The line shows y = 0.1/x. (0.37 MB PDF) [file pone.0007943.s001.pdf]

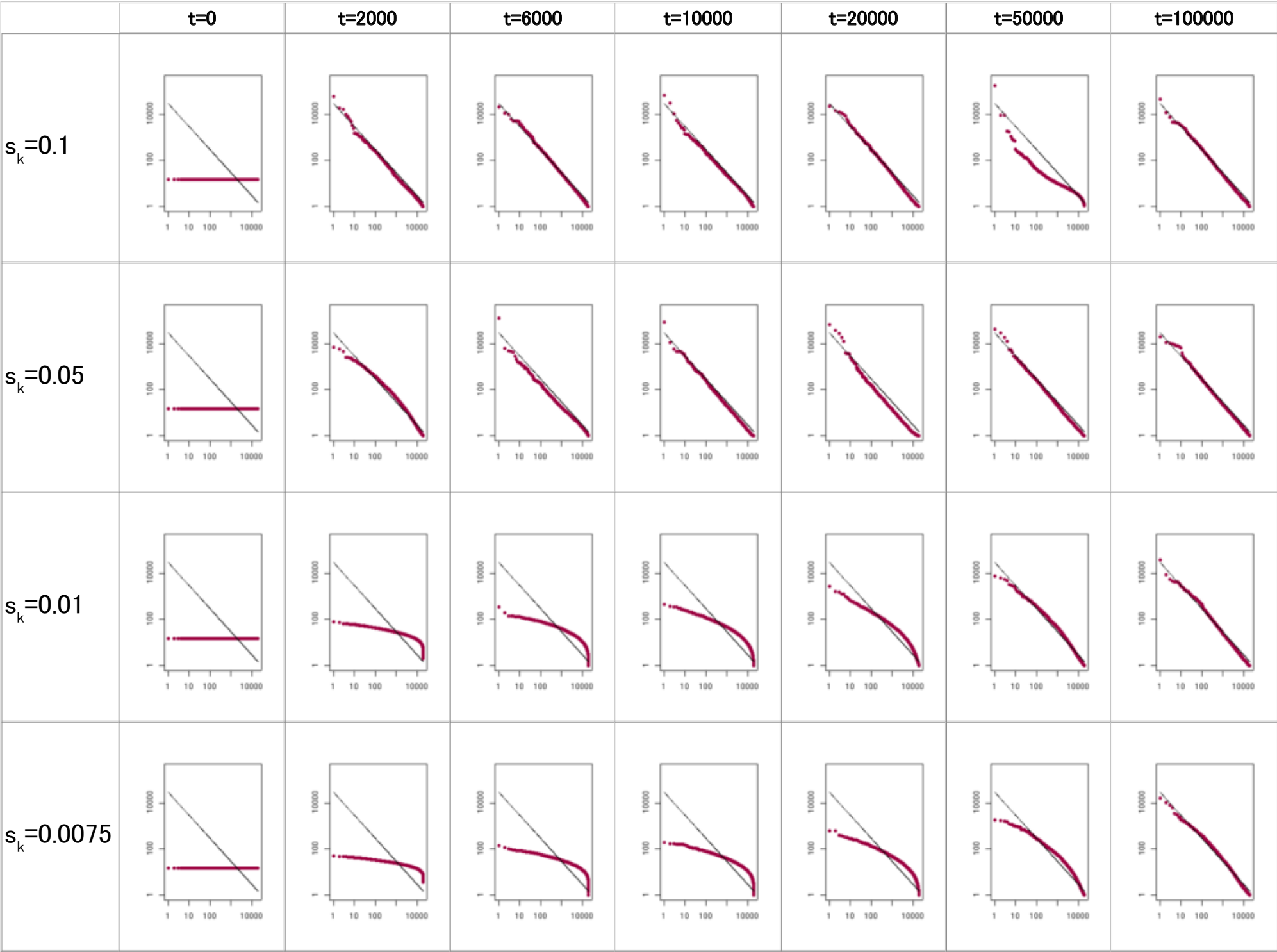

Supplement: Figure S2 — Time development of hypothetical mRNA abundance generated by Monte Carlo simulations of the refined neutral model (L = 1.0) Other model parameters were: M = 20,000, N = 300,000. The line shows y = 0.1/x. (0.39 MB PDF) [file pone.0007943.s002.pdf]
